# The effect of rest redistribution on kinetic and kinematic variables during the hang pull

**DOI:** 10.1371/journal.pone.0299311

**Published:** 2024-02-26

**Authors:** David Meechan, John J. McMahon, Timothy J. Suchomel, Paul Comfort

**Affiliations:** 1 Human Performance Laboratory, Directorate of Psychology and Sport, University of Salford, Salford, Greater Manchester, United Kingdom; 2 Department of Elite Training Science and Technology Department, Hong Kong Sports Institute, Hong Kong, China; 3 Sport Science and Sport Medicine Centre, Singapore Sport Institute, Singapore, Singapore; 4 Department of Human Movement Sciences, Carroll University, Waukesha, Wisconsin, United States of America; 5 Strength and Power Research Group, School of Medical and Health Sciences, Edith Cowan University, Joondalup, Western Australia; University of Belgrade: Univerzitet u Beogradu, SERBIA

## Abstract

The aim of this study was to compare the effects of rest redistribution (RR) on kinetics and kinematics during the hang pull (HP). Twenty-one male athletes (age 29.5 ± 4.3 years, height 1.78 ± 0.07 m, body mass 75.17 ± 11.11 kg, relative one repetition maximum [1RM] power clean [PC] 1.17 ± 0.14 kg.kg^-1^) performed the HP using 140% of 1RM PC with 3 traditional sets of 6 repetitions (TS), 9 sets of 2 repetitions with RR [45s rest after 2 repetitions] (RR_45_) and 6 sets of 3 repetitions with RR [72s rest after 3 repetitions] (RR_72_). Peak velocity (PV) was higher during RR_72_ (1.18 ± 0.11 m.s^-1^) compared to RR_45_ (1.14 ± 0.11 m.s^-1^) for the average of 18 repetitions (*p* = 0.025, *g* = 0.36). There was a main effect for set configuration with greater peak force (PF) (*p* < 0.001, *g* = 0.14) during RR_72_ compared to RR_45,_ with greater PV and impulse (*p* < 0.001, *g* = 0.19–0.36) during RR_72_ compared to RR_45_. There was also greater peak velocity maintenance (PVM) (*p* = 0.042, *g* = 0.44) for RR_72_ compared to RR_45._ There were no significant or meaningful differences (*p* > 0.05, *g* = 0.00–0.59) between configurations for any other variables. Rest redistribution protocols did not result in significantly or meaningfully greater kinetics or kinematics during the HP when compared to a TS protocol; although performing RR_72_ resulted in higher PF, PV, and impulse, with improved PVM compared to RR_45._

## Introduction

Weightlifting pulling derivatives are regularly performed in resistance training as they emphasize rapid force development, however, omitting the catch phase allows for loading greater than an athlete’s one repetition maximum (1RM) power clean (PC) [1–6]. Recently, researchers demonstrated that the hang pull (HP) results in consistently higher mean system force, power, and velocity across multiple loads, with greater peak system velocity, barbell velocity, peak power, and net impulse compared to the pull from the knee with loads ≥100% 1RM PC [1]. As such, this may be a superior exercise to develop these characteristics compared to weightlifting derivatives that do not emphasize the utilization of the stretch-shortening cycle (SSC [e.g., the pull from the knee]), especially at loads ≥100% 1RM PC [1].

When performing multiple repetitions consecutively (i.e., traditional sets [TS]) there is a general trend of a progressive decrease in barbell velocity and displacement in weightlifting derivatives [5–7], leading to reductions in power output [7], with an increased rating of perceived exertion (RPE) [5]. To maintain kinetics and kinematics during weightlifting exercises, additional intra-set rest periods, termed ‘cluster sets’ are regularly prescribed [4–8]. While ‘cluster sets’ are an excellent method of minimizing intra-set fatigue, the additional time taken to complete training (due to the added intra-set rest between clusters) is not always feasible in time constrained training sessions.

The reallocation of overall rest periods, termed rest-redistribution (RR), for the purpose of creating more frequent sets (utilizing some of the between-set rest time within the sets), has been recommended to minimize fatigue [4, 5, 9] and maintain kinetics and kinematics [4–6, 8]. This approach, which does not extend training duration, may prove advantageous in practical settings.

Jukic and Tufano [4] suggested that when compared to TS training, RR may only be beneficial when the sets are highly fatiguing, therefore, RR implemented across various loads or different barbell displacements may yield different findings [10]. Researchers have demonstrated that cluster sets and RR are generally superior at maintaining velocity, power, barbell displacement, with lower RPE than TS structures across various loads (80–120% 1RM PC) during weightlifting movements [4, 6–9]. As greater power has been associated with lower RPE scores in weightlifting movements [9], non-fatiguing set structures are likely beneficial.

To date, no researchers have investigated the effects of RR on the kinetics and kinematics of the hang pull (HP). As the HP omits the catch [1] performing loads >100% 1RM PC are achievable [1, 3]. While PC technique at 80%1RM has been shown to deteriorate across consecutive repetitions [8], this deterioration may not be evident during the HP due to it being a less complex exercise (as it has a shorter range of motion and no catch phase), and therefore, kinetics and kinematics may be better maintained across multiple repetition and sets. However, further investigation is warranted to investigate this at loads >100% 1RM PC as this may result in different findings. This may allow for an alternative stimulus for the prescription of a high force and semi-ballistic training modality with higher training volumes within a training session.

The purpose of this study was to investigate the effects of RR on changes in kinetics (force, impulse) and kinematics (system velocity), propulsion duration, peak velocity maintenance (PVM), and RPE at 140% 1RM PC during the HP over multiple repetitions and sets. This load was selected as it was the load that previously maximized force during the HP and is likely the most fatiguing, as it is the heaviest load previously examined [1]. It was hypothesized that both RR protocols containing shorter but more frequent rest periods would result in a greater force and system velocity over multiple repetitions and sets of the HP, while also resulting in lower propulsion duration and RPE compared to TS configurations, in agreement with previous findings [5–7, 11].

## Materials and methods

A within-subject repeated-measures research design was used; whereby kinematic (peak and mean system velocity) and kinetic (peak and mean force and impulse) variables, and phase duration were determined, from the force-time data, during the propulsion phase of the HP. Prior to the experimental trials, subjects visited the strength and conditioning facility on two occasions, at the same time of day (5–7 days apart), to establish 1RM PC reliability following a previously used protocol [2]. Additionally, familiarisation with 0–10 OMNI-RES scale: a resistance training specific RPE scale [12] was also undertaken.

**Fig 1:**
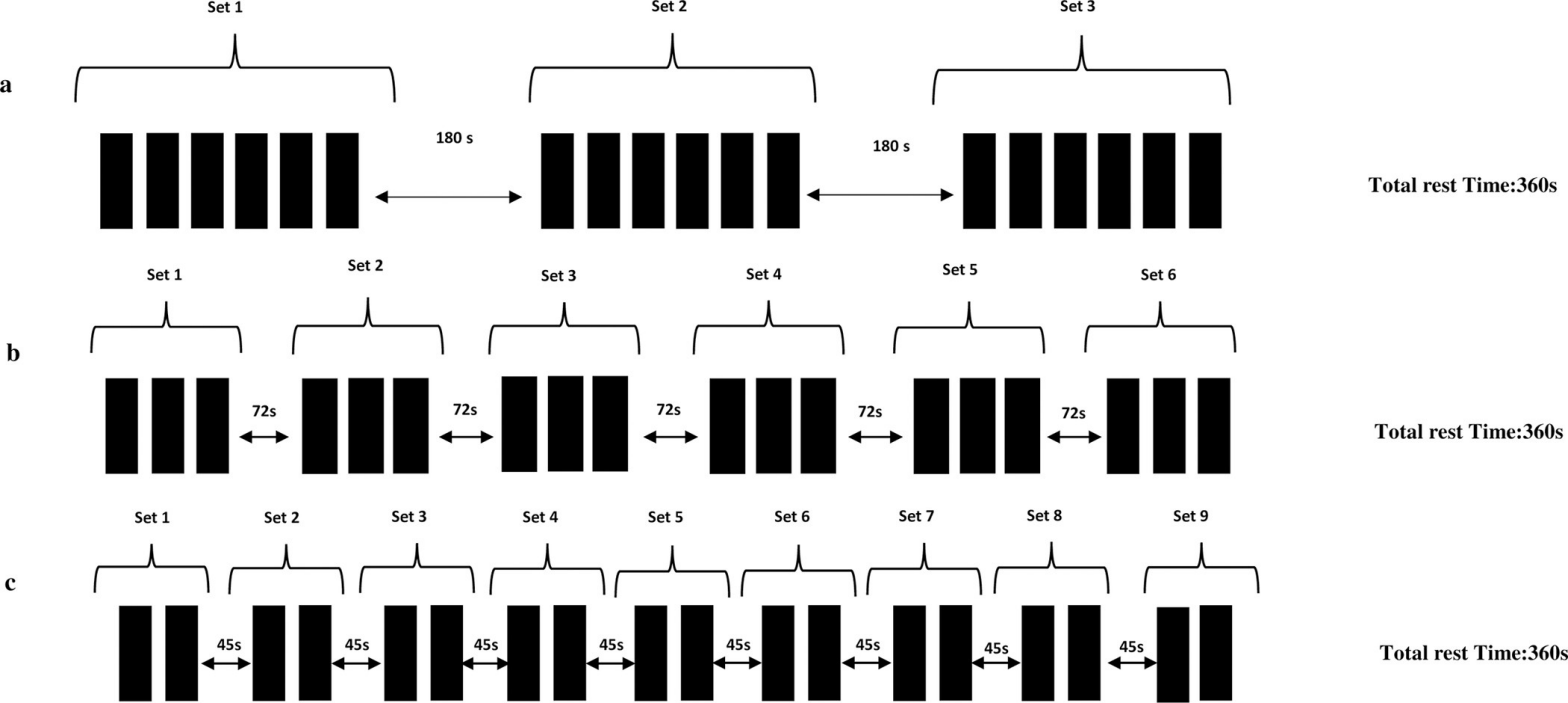
Set structure protocols.

For the experimental trials, each subject performed the HP exercise for one of the following randomly assigned and counterbalanced protocols across three sessions: 3 sets of 6 repetitions using 140% (TS) of 1RM PC with 180 seconds of inter-set rest (2 x 180 = 360 s of total rest), RR protocols of 9 sets of 2 repetitions using 140% (RR_45_) of PC 1RM with 45 seconds of inter-set rest (8 x 45 = 360 s of total rest), and 6 sets of 3 repetitions using 140% (RR_72_) with 72 seconds of inter-set rest (5 x 72 = 360 s of total rest). All subjects completed all 18 repetitions in all 3 experimental sessions (Fig 1).

### Subjects

An a priori sample size calculation was performed, identifying a minimum sample of 18 subjects was required, for a statistical power of 0.80 at an alpha level of 0.05, based on an average effect size of 0.60 for differences in peak velocity (specific values are not provided but illustrated in forest plots) effect sizes (Cohen’s *d* = 0.60 [range = 0.20–1.10]) [13]. Twenty-one male subjects from various teams and individual sports including rugby, soccer, track cycling, martial arts, and athletics (long jump, javelin) (age = 29.50 ± 4.30. years, height = 1.78 ± 0.07 m, body mass = 75.17 ± 11.11 kg, resistance training experience = 7.50 ± 1.48 years, relative 1RM PC = 1.17 ± 0.15 kg.kg^-1^) who participated in regular resistance training (≥2 x week), including experience with weightlifting derivatives, volunteered to participate in this study, and were recruited from 20^th^ November 2021 to 31^st^ March 2022. Subjects were free from injury, provided written informed consent, and were requested to perform no strenuous activity during the 48 hours before testing, maintain their normal dietary intake before each session, and to attend testing sessions in a hydrated state. Subjects were all in a strength and power phase during in season of their respective sports. This investigation received ethical approval from the University of Salford’s Ethics Committee and conformed to the principles of the World Medical Association’s Declaration of Helsinki and no author had access to information that could identify individual participants during or after data collection. The individual in this manuscript has given written informed consent (as outlined in PLOS consent form) to publish these case details.

### One repetition maximum power clean testing

Subjects performed a 5-minute dynamic warm-up that consisted of body weight squats, lunges, and dynamic stretching. Three sub-maximal PC efforts were performed with decreasing volume (6, 4, and 2 repetitions) and increasing loads of approximately 50, 70, and 90% 1RM PC (matched to the volume) before commencing their first 1RM attempt, in line with previous research [1, 2]. The 1RM PC for each subject was then determined within five attempts (interspersed by 3–4 minutes of rest) by gradually increasing the load (2.5–5.0 kg increments) until a failed attempt occurred. All PC attempts began with the load on the lifting platform and ended with the barbell caught on the anterior deltoids in a semi-squat position above parallel (visually monitored and any attempt caught below this was disallowed). The greatest load achieved across the two sessions was used to calculate the loads used for the HP during the TS and RR sets. An accredited strength and conditioning coach supervised all sessions.

### Experimental testing

**Fig 2:**
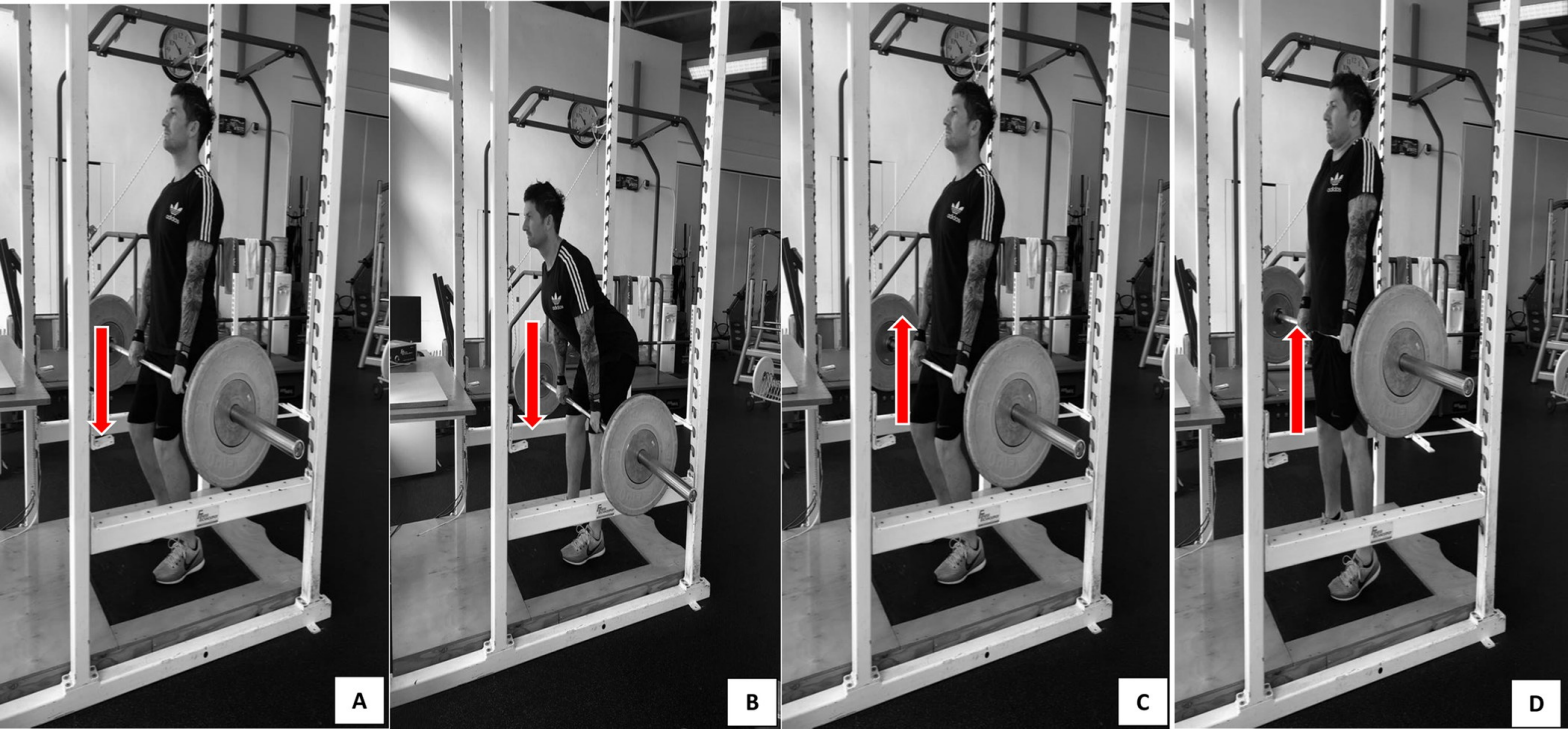
Sequence of hang pull.

The subjects performed the HP with loads based off their 1RM PC. The warm-up consisted of the same dynamic warm-up as the 1RM testing sessions, after which the subjects’ performed sets of 5, 4, 3, 2 and 1 repetition at 50, 60, 70, 80 and 90% of 140% of their 1RM PC in line with previous research [1, 2]. The barbell was placed on the safety bars of the power cage during rest periods to prevent fatigue. Once the body was stabilized (verified by observing the subject and force-time data), the lift was initiated with the countdown “3, 2, 1, go,” and all subjects were instructed to exert maximal intent during each repetition. All lifts were performed in a power cage with an integrated force plate (400 Series, Fitness Technology, Adelaide, Australia) sampling at 600 Hz, interfaced with a desktop computer and ballistic measurement software (Version 1). Verbal encouragement was provided and lifting straps were used throughout testing. Prior to the HP (Fig 2) subjects started each movement in a standing position, with their knees slightly bent and the barbell positioned at the midthigh, with the subjects holding the barbell at arm’s length (Fig 2). Subjects then performed a countermovement by flexing at the hip, while maintaining their knee angle and lowering the barbell to a position just above their patella. Upon reaching this position, the subjects immediately transitioned back to the mid-thigh position by flexing their knees and extending their hips to bring their torso to an upright position followed immediately by a rapid triple extension of the hip, knee, and ankle (plantar flexion) and a shrug that moved the barbell in a vertical plane while maintaining elbow extension (i.e., second pull) in one continuous movement [1].

### Force-time data collection

Prior to the onset of the pull, subjects were instructed to remain stationary in the 2^nd^ pull position [10] for 1 second to allow for determination of the system weight (body mass plus barbell weight) [14, 15]. Vertical ground reaction force (VGRF) data were averaged across the first second while the subjects stood still (this average value represented the system weight), and a force threshold was calculated from the VGRF during this same time period. Specifically, the standard deviation of the VGRF across the first second was calculated and then multiplied by 5, and the resultant value represented the force threshold used to determine the onset of the HP [14]. During the HP, the onset of movement was deemed to have occurred 30 ms before the VGRF was exceeded and reduced by the force threshold, respectively [14]. Velocity of the system (barbell plus body) was calculated from VGRF force-time data. The acceleration-time record (subtracting system weight from VGRF and then dividing this by system mass on a sample-by sample basis) was numerically integrated using the trapezoid rule to yield the velocity-time record [14, 15]. Net VGRF was integrated with respect to time (also using the trapezoid rule) to obtain the net impulse. All force-time variables were further analysed during the propulsion phase only. The propulsion phase was deemed to have commenced when system velocity was >0.01 m·s^-1^ and finished at peak velocity, which coincided with the end of the pull [16–18]. Peak (PF) and mean force (MF), peak system velocity (PV), mean system velocity (MV), impulse (IMP), and phase duration were attained during the propulsion phase [16–18]. Since the number of repetitions per set differed between RR_45_ (2 repetitions per set), RR_72_ (3 repetitions per set), and TS (6 repetitions per set), RR_45_ sets 1 to 3, 4 to 6 and 7 to 9 and RR_72_ sets 1 to 2, 3 to 4, and 5 to 6 were grouped together to create ‘3 sets’ for comparison purposes. Raw vertical force-time data for each trial was exported as text files and analysed using a customized Excel spreadsheet (version 2016; Microsoft Corp., Redmond, WA, USA). For each protocol, the absolute values for PF, MF, PV, MV, IMP, and propulsion duration were each combined and averaged across all 18 repetitions for each configuration. This was also performed for the ‘3 sets of 6’ within each protocol. Peak velocity maintenance was calculated with the following equation: Maintenance set = 100 -[mean set–repetition_max_] *100 [5]. This approach was used rather than comparing the first and last repetitions, as the first repetition is not always the fastest and the last repetition is not always the slowest [5, 7].

### Rating of perceived exertion

Familiarisation of the RPE scale took place during the first 1RM reliability testing session. During the experimental sessions, RPE (0 = no effort, 10 = maximal effort) was obtained after the 6^th^, 12^th^, and 18^th^ repetitions. In line with previous research investigating weightlifting movements, these three RPE scores were also averaged together to create an average RPE for each protocol and after each set of six [4, 5].

### Statistical analyses

Statistical analyses were performed using SPSS version 26 (SPSS, Chicago, IL, USA). Distribution of data were analysed by using the Shapiro-Wilks test of normality. Reliability for 1RM PC was assessed using two-way mixed intraclass correlation (ICC), with associated 95% confidence intervals (CI) and typical error expressed as a coefficient of variation percentage (CV%)]. The ICC was interpreted as poor (< 0.50), moderate (0.50–0.74), good (0.75–0.90), and excellent (>0.90), based on the lower bound 95% CI [19]. Acceptable within-session variability was classified as <10% [20]. Differences between configurations were determined using repeated-measures analysis of variance (ANOVA) with Bonferroni post hoc analysis or Friedman’s tests with multiple Wilcoxon’s tests (including Bonferroni correction for multiple comparison to reduce the risk of Type 1 errors) for data that were not normally distributed. Differences between TS, RR_45_, and RR_72_ within each set was examined using a series of two-way repeated-measures ANOVA. When a significant main effect or interaction was determined, Bonferroni post-hoc tests were conducted. Sphericity could not be assumed by the Mauchly test (*p* > 0.05) for all variables, and therefore, Greenhouse-Geisser adjustment was used. Standardized differences were calculated using Hedges’ *g* effect sizes, as previously described [16] and interpreted as trivial (≤ 0.19), small (0.20–0.59), moderate (0.60–1.19), large (1.20–1.99), and very large (2.0–4.0) [21]. An *a priori* alpha level was set at *p* ≤ 0.05.

## Results

Power clean 1RM performances were highly reliable (ICC = 0.98, [95% CI = 0.85–0.99], %CV = 2.0% [1.3–3.1%] between session 1 (87.29 ± 14.24 kg) and session 2 (84.81 ± 14.74 kg). The HP kinetics and kinematics assessed in the study have been previously reported to demonstrate moderate to excellent reliability and acceptable variability [1].

### Kinetics and kinematics differences between protocols across 18 repetitions

There was significantly greater (*p* = 0.025, *g* = 0.36), PV during RR_72_ compared to RR_45_ (Table 1). There were no significant or meaningful differences (*p* > 0.05, *g* = 0.00–0.59) between configurations for any other variables (Table 1 and S1A, S1B and S1D–S1F Fig).

**Table 1:** Means ± standard deviations (95% confidence intervals) for propulsive kinetics and kinematics, phase duration, peak velocity decline, peak velocity maintenance and RPE for all 18 repetitions averaged within each protocol during the hang pull at 140% 1RM.

Variable	TS (3x6) 18 repetitions (95%CI)	RR_45_ (9x2) 18 Repetitions (95% CI)	RR_72_ (6x3) 18 Repetitions (95% CI)	*p* value, (*g*)		
				TS vs RR_45_	TS vs RR_72_	RR_45_ vs RR_72_
**Peak Force (N)**	3150 ± 495 (2925–3375)	3099 ± 443 (2898–3301)	3162 ± 476 (2945–3379)	*p* > 0.05 (0.12)	*p* > 0.05 (0.03)	*p* > 0.05 (0.13)
**Mean Force (N)**	2681 ± 461 (2471–2891)	2662 ± 459 (2453–2871)	2686 ± 451 (2481–892)	*p* > 0.05 (0.04)	*p* > 0.05 (0.01)	*p* > 0.05 (0.05)
**Peak Velocity (m.s-** ^**1**^ **)**	1.16 ± 0.09 (1.12–1.21)	1.14 ± 0.11 (1.09–1.19)	1.18 ± 0.11 (1.13–1.23)	*p* > 0.05 (0.20)	*p* > 0.05 (0.20)	*p* = 0.025** (0.36)
**Mean Velocity (m.s-** ^**1**^ **)**	0.55 ± 0.06 (0.52–0.58)	0.55 ± 0.07 (0.52–0.58)	0.56 ± 0.07 (0.52–0.59)	*p* > 0.05 (0.00)	*p* > 0.05 (0.15)	*p* > 0.05 (0.14)
**Impulse (N.s)**	231 ± 38 (214–248)	226 ± 38 (209–243)	233 ± 37 (216–249)	*p* > 0.05 (0.13)	*p* > 0.05 (0.05)	*p* > 0.05 (0.18)
**Phase Duration (s)**	0.493 ± 0.05 (0.469–0.517)	0.497 ± 0.07 (0.465–0.529)	0.495 ± 0.07 (0.463–0.527)	*p* > 0.05 (0.06)	*p* > 0.05 (0.03)	*p* > 0.05 (0.03)
**Velocity Maintenance (%)**	90 ± 5 (87.6–92.3)	88 ± 5 (86.-90.1)	91 ± 5 (88.5–93.4)	*p* > 0.05 (0.39)	*p* > 0.05 (0.20)	*p* > 0.05 (0.59)
**RPE**	7.07 ± 1.03 (6.60–7.54)	6.98 ± 0.80 (6.62–7.35)	6.87 ± 0.78 (6.53–7.23)	*p* > 0.05 (0.10)	*p* > 0.05 (0.21)	*p* > 0.05 (0.14)

**Denotes significant differences between set configurations

### Set by set with interaction

There was a significant main effect for configuration on PF, with significantly greater PF during RR_72_ compared to RR_45_ (Table 2)_._ There was a significant main effect for configuration on PV, with significantly greater PV during RR_72_ compared to RR_45_ (Table 2). There was a significant main effect for configuration on IMP, with greater IMP during RR_72_ compared to RR_45_ (Table 2). There was a significant main effect for configuration on PVM, with significantly greater PVM for RR_72_ compared to RR_45_ (Table 2)_._ There was a significant main effect of set, with significantly lower RPE for set 1 compared to set 2, and significantly lower RPE for set 1 compared to set 3, with significantly lower RPE for set 2 compared to set 3 (Table 2). There were no other significant differences for configuration, set or set x configuration (Table 2).

**Table 2:** Results of repeated measures two-way analysis of variance with interaction effect.

Variable	ANOVA Result	*p*-value	Comparison
**ANOVA- Configuration**			
**Peak Force (N)**	*F* (1.82,109) = 5.813, *p* = 0.005	***p* < 0.001** *	RR_72_ significantly greater than vs. RR_45_
**Mean Force (N)**	*F* (2,120) = 1.358, *p* = 0.261	*p* ≥ 0.296	No significant differences between protocols
**Peak Velocity (m.s-** ^**1**^ **)**	*F* (2,120) = 6.372, *p* = 0.002	***p* < 0.001** *	RR_72_ significantly greater than RR_45_
**Mean Velocity (m.s-** ^**1**^ **)**	*F* (2,120) = 0.225, *p* = 0.799	*p* = 1.000	No significant differences between protocols
**Impulse (N.s)**	*F* (1.80, 107.9) = 5.876, *p* = 0.005	***p* < 0.001** *	RR_72_ significantly greater than RR_45_
**Phase Duration (s)**	*F* (2, 120) = 0.315, *p* = 0.730	*p* = 1.000	No significant differences between protocols
**Velocity Maintenance (%)**	*F* (2, 120) = 4.179, *p* = 0.018	***p* = 0.042** *	RR_72_ significantly greater than RR_45_
**RPE**	*F* (1.81, 109) = 1.273, *p* = 0.282	*p* ≥ 0.407	No significant differences between protocols
**ANOVA-Set**			
**Peak Force (N)**	*F* (2,60) = 0.040, *p* = 0.961	*p* = 1.000	No significant differences between sets
**Mean Force (N)**	*F* (2,60) = 0.004, *p =* 0.996	*p* = 1.000	No significant differences between sets
**Peak Velocity (m.s-** ^**1**^ **)**	*F* (2,60) = 0.091, *p =* 0.913	*p* = 1.000	No significant differences between sets
**Mean Velocity (m.s-** ^**1**^ **)**	*F* (2, 60) = 0.101, *p* = 0.904	*p* = 1.000	No significant differences between sets
**Impulse (N.s)**	*F* (2, 60) = 0.017, *p* = 0.983	*p* = 1.000	No significant differences between sets
**Phase Duration (s)**	*F* (2, 60) = 0.036, *p* = 0.965	*p* = 1.000	No significant differences between sets
**Velocity Maintenance (%)**	*F* (2, 60) = 1.078, *p* = 0.347	*p* ≥ 0.545	No significant differences between sets
**RPE**	*F* (2, 60) = 15.763, *p* < 0.001	***p* = 0.010** * ***p* < 0.001** * ***p* = 0.041** *	Set 2 significantly greater than Set 1 Set 3 significantly greater than Set 1 Set 3 significantly greater than 2
**ANOVA- Set x Configuration**			
**Peak Force (N)**	*F* (3.63,109) = 0.996	*p* = 0.408	No significant differences
**Mean Force (N)**	*F* (4,120) = 0.696	*p* = 0.596	No significant differences
**Peak Velocity (m.s-** ^**1**^ **)**	*F* (4,120) = 1.146,	*p* = 0.338	No significant differences
**Mean Velocity (m.s-** ^**1**^ **)**	*F* (4,120) = 1.099	*p* = 0.360	No significant differences
**Impulse (N.s)**	*F* (3.597, 107.9) = 1.103	*p* = 0.357	No significant differences
**Phase Duration (s)**	*F* (4, 120) = 1.395	*p* = 0.240	No significant differences
**Velocity Maintenance (%)**	*F* (4, 120) = 0.905	*p* = 0.463	No significant differences
**RPE**	*F* (3.622, 109) = 0.357	*p* = 0.821	No significant differences

*Denotes significant differences

### Set by set comparison (within configuration)

Peak force was significantly greater in set 3 compared to set 1 during the TS configuration (Table 3). During the RR_72,_ PF was also significantly greater in set 2 and set 3 compared to set 1 (Table 3). There was significantly greater MV during RR_45_ in set 1 compared to set 3 (Table 3). There was significantly greater phase duration during RR_72_ in set 1 compared to set 3 (Table 3). The RPE was significantly lower for the TS configuration during set 1 compared to set 2 and set 3, and significantly lower RPE during set 2 compared to set 3 (Table 3). The RPE was also significantly lower for the RR_45_ configuration during set 1 compared to set 2 and set 3, and significantly lower RPE during set 2 compared to set 3 (Table 3). Similarly, RPE was significantly lower during the RR_72_ configuration during set 1 compared to set 2 and set 3, and significantly lower RPE during set 2 compared to set 3 (Table 3). There were no significant or meaningful differences between configurations for any other variables (S1–S3 Figs; Table 3).

**Table 3:** Between set comparisons of kinetics and kinematics, phase duration and rate of perceived exertion for each set of 6 repetitions for traditional sets (3 x 6), rest redistribution (9 x 2 and 6 x 3) data presented as means ± standard deviations (95% confidence intervals).

Variable	Set 1 (95%CI)	Set 2 (95%CI)	Set 3 (95%CI)	*p* value Hedges (*g)*		
				Set 1 vs Set 2	Set 1 vs Set 3	Set 2 vs Set 3
**Traditional Sets 3 x 6**						
**Peak Force (N)**	3108 ± 478 (2890–3326)	3157 ± 500 (2929–3385)	3185 ± 517 (2949–3420)	*p* > 0.05 (0.10)	*p* = 0.013** (0.15)	*p* > 0.05 (0.05)
**Mean Force (N)**	2672 ± 454 (2465–2879)	2677 ± 467 (2464–2889)	2695 ± 469 (2681–2908)	*p* > 0.05 (0.01)	*p* > 0.05 (0.05)	*p* > 0.05 (0.04)
**Peak Velocity (m.s-** ^**1**^ **)**	1.16 ± 0.11 (1.11–1.21)	1.16 ± 0.10 (1.11–1.20)	1.18 ± 0.09 (1.14–1.22)	*p* > 0.05 (0.00)	*p* > 0.05 (0.20)	*p* > 0.05 (0.21)
**Mean Velocity (m.s-** ^**1**^ **)**	0.55 ± 0.06 (0.52–0.58)	0.54 ± 0.06 (0.52–0.57)	0.56 ± 0.07 (0.53–0.60)	*p* > 0.05 (0.16)	*p* > 0.05 (0.16)	*p* > 0.05 (0.33)
**Impulse (N.s)**	229 ± 37 (212–245)	230 ± 36 (212–248)	234 ± 39 (216–252)	*p* > 0.05 (0.03)	*p* > 0.05 (0.13)	*p* > 0.05 (0.10)
**Phase Duration (s)**	0.49 ± 0.06 (0.47–0.52)	0.49 ± 0.05 (0.47–0.52)	0.49 ± 0.05 (0.47–0.52)	*p* > 0.05 (0.02)	*p* > 0.05 (0.05)	*p* > 0.05 (0.04)
**RPE**	6.50 ± 1.18 (5.96–7.04)	7.12 ± 1.05 (6.64–7.60)	7.60 ± 1.04 (7.12–8.07)	*p* = 0.001**(0.54)	*p* = 0.006**(0.97)	*p* = 0.015** (0.45)
**Velocity Maintenance (%)**	94.2 ± 3 (92.9–95.6)	94.5 ± 3.1 (93.1–95.9)	93.5 ± 3.2 (92.1–95)	*p* > 0.05 (0.10)	*p* > 0.05 (0.22)	*p* > 0.05 (0.31)
**Rest Redistribution 9 x 2**						
**Peak Force (N)**	3108 ± 423 (2915–3300)	3089 ± 457 (2882–3297)	3101 ± 455 (2893–3308)	*p* > 0.05 (0.04)	*p* > 0.05 (0.02)	*p* > 0.05 (0.03)
**Mean Force (N)**	2677 ± 437 (2478–2876)	2655 ± 474 (2439–2871)	2655 ± 469 (2441–2869)	*p* > 0.05 (0.05)	*p* > 0.05 (0.05)	*p* > 0.05 (0)
**Peak Velocity (m.s-** ^**1**^ **)**	1.16 ± 0.11 (1.11–1.21)	1.13 ± 0.11 (1.08–1.18)	1.14 ± 0.12 (1.08–1.19)	*p* > 0.05 (0.27)	*p* > 0.05 (0.17)	*p* > 0.05 (0.09)
**Mean Velocity (m.s-** ^**1**^ **)**	0.56 ± 0.07 (0.53–0.60)	0.55 ± 0.07 (0.51–0.58)	0.54 ± 0.07 (0.51–0.57)	*p* > 0.05 (0.14)	*p* = 0.046**(0.28)	*p* > 0.05 (0.14)
**Impulse (N.s)**	229 ± 36 (213–246)	224 ± 40 (206–243)	224 ± 39 (207–242)	*p* > 0.05 (0.13)	*p* > 0.05 (0.13)	*p* > 0.05 (0.00)
**Phase Duration (s)**	0.49 ± 0.06 (0.46–0.52)	0.50 ± 0.07 (0.47–0.53)	0.50 ± 0.08 (0.46–0.54)	*p* > 0.05 (0.15)	*p* > 0.05 (0.14)	*p* > 0.05 (0.00)
**RPE**	6.24 ± 0.82 (5.87–6.61)	7.05 ± 0.88 (6.65–7.45)	7.67 ± 0.86 (7.28–8.06)	*p* < 0.001**(0.93)	*p* < 0.001**(1.67)	*p* = 0.001** (0.70)
**Velocity Maintenance (%)**	91.8 ± 4.9 (89.5–94)	93.2 ± 3.3 (91.7–94.8)	93.3 ± 4 (91.5–95.1)	*p* > 0.05 (0.12)	*p* > 0.05 (0.34)	*p* > 0.05 (0.05)
**Rest Redistribution 6 x 3**						
**Peak Force (N)**	3130 ± 487 (2908–3352)	3175 ± 473 (2906–3391)	3181 ± 472 (2966–3396)	*p* = 0.016**(0.09)	*p* = 0.005**(0.10)	*p* > 0.05 (0.01)
**Mean Force (N)**	2667 ± 456 (2459–2874)	2691 ± 452 (2485–2897)	2701 ± 449 (2497–2906)	*p* > 0.05 (0.05)	*p* > 0.05 (0.07)	*p* > 0.05 (0.02)
**Peak Velocity (m.s-** ^**1**^ **)**	1.18 ± 0.12 (1.14–1.23)	1.17 ± 0.11 (1.12–1.23)	1.18 ± 0.12 (1.13–1.23)	*p* > 0.05 (0.09)	*p* > 0.05 (0.00)	*p* > 0.05 (0.09)
**Mean Velocity (m.s-** ^**1**^ **)**	0.55 ± 0.07 (0.52–0.59)	0.55 ± 0.08 (0.52–0.59)	0.56 ± 0.07 (0.52–0.59)	*p* > 0.05 (0.00)	*p* > 0.05 (0.14)	*p* > 0.05 (0.13)
**Impulse (N.s)**	233 ± 36 (217–250)	232 ± 36 (215–248)	233 ± 38 (215–250)	*p* > 0.05 (0.03)	*p* > 0.05 (0.03)	*p* > 0.05 (0.00)
**Phase Duration (s)**	0.51 ± 0.07 (0.47–0.54)	0.50 ± 0.08 (0.46–0.53)	0.49 ± 0.06 (0.46–0.51)	*p* > 0.05 (0.13)	*p* = 0.003**(0.30)	*p* > 0.05 (0.14)
**RPE**	6.20 ± 0.75 (5.85–6.53)	6.88 ± 0.92 (6.46–7.30)	7.55 ± 0.89 (7.14–7.95)	*p* = 0.001**(0.79)	*p* < 0.001**(1.61)	*p* = 0.002** (0.73)
**Velocity Maintenance (%)**	93.4 ± 4.5 (91.4–95.4)	94.6 ± 3.0 (93.2–96)	95.1 ± 2.1 (94.1–96.1)	*p* > 0.05 (0.31)	*p* > 0.05 (0.48)	*p* > 0.05 (0.20)

** denote significant differences between sets.

## Discussion

The primary aim of this study was to compare the effects of RR protocols on kinetics, kinematics, and perceptual fatigue (i.e., RPE) during the HP performed using 140% 1RM PC, compared to a TS protocol. When compared across the average of all 18 repetitions, incorporating RR protocols did not result in different PF, MF, MV, IMP, propulsion phase duration, PVM, or RPE across protocols. However, the RR_72_ protocol resulted in greater PV compared to RR_45_ averaged across 18 repetitions, although these were not significantly different compared to the TS protocol (Table 1)_._ Therefore, shorter more frequent rest periods during the HP may not be required to maintain force-time characteristics across a multiple set protocol in this cohort.

Across the average of 18 repetitions, there were no significant differences between RR and TS configurations for any kinetic and kinematic variables. Therefore, weightlifting pulling derivatives such as the HP may be able to be performed with much greater set and session volumes than other weightlifting movements regardless of load. One argument against performing sets with higher repetition schemes centres on the fact that weightlifting technique becomes impaired with increasing fatigue [6]. However, based upon the findings of this study, it appears possible to perform the HP with higher intensities for higher volumes per set, while maintaining kinetics and kinematics, which could lead to a different training stimulus being applied compared to sets with lower repetitions at the same load. Maintaining mechanical outputs is imperative for inducing the most appropriate training stimulus in weightlifting derivatives, which ultimately leads to a performance improvement [6].

Strength–speed may be described as the ability to quickly perform a movement against a relatively large external load and is assessed in terms of mass lifted [22], and ultimately, mechanical power may be improved by performing a mixed methods approach by training with both high velocity and high force exercises [23]. Weightlifting pulling derivatives such as the HP allow the athlete to emphasise the higher force (strength-speed) aspect of the force-velocity curve [22, 24]. Regardless of TS or RR protocols, subjects in the study achieved average peak velocities between 1.14 and 1.18 m s^−1^ and mean velocities between 0.55 and 0.56 m s^−1^ across 18 repetitions at 140% 1RM PC. This indicates the ability to accelerate the barbell with moderately high velocities across multiple sets and repetitions with minimal performance decrement (Table 1, S1C and S1D Fig). Although direct comparisons may not be possible due the differences in measurement device and exercise selection, it may be possible to perform a higher volume of repetitions during the HP in a ‘strength-speed’ zone, which may provide a unique training stimulus without compromising technique due to the velocities noted in a recent review [22]. However, caution is warranted when comparing barbell and system measurements [25], as it is imperative to acknowledge that each exercise has a distinct %1RM-velocity profile, and thus generalizing velocity zones across different exercises may not be valid [26].

Previously, researchers have suggested that mechanical power would be determined by differences in movement velocity during different set structures [27, 28]. Across the average of 18 repetitions, it is likely that the RR_72_ protocol would have shown a greatest propulsion power output compared to both RR_45_ and TS as there was slightly greater force and velocity during the RR_72_ configuration (Table 1). Interestingly, when comparing the differences between sets (Table 3), the greatest forces and velocities were observed in set 3 during the TS protocol, however, during RR_45_, this was evident in set 1, perhaps demonstrating that no fatigue occurred. These observations are likely related to un-racking and re-racking of the bar, and therefore, coaches should play careful consideration to this when selecting set configurations as it may be exercise/load specific. It may be possible that the athletes adopted a pacing strategy during the TS protocol in particular [29], and perhaps the subjects put in more effort in the last set of the TS as they knew that this was the final set.

Interestingly, during all the TS and RR_45_ sets, the second repetition was always faster than the 1^st^ repetition (S1C Fig). It could be assumed that the inclusion of multiple RR protocols would allow for greater velocity compared to TS protocols, due to the short rest period preceding the next repetition (e.g., repetition 3 of RR_45_ vs. repetition 3 of TS [there is 45s second IRR between the 2^nd^ and 3^rd^ repetition]). However, this was not always evident with repetitions 5, 11, and 17, the first repetition in each of sets of 2 being slower than the corresponding repetition during the RR_72_ configuration, which highlights the requirement for the practitioner to carefully consider optimal set and repetition structures to maximize kinetic and kinematic output within a training session. Further, Meechan et al. [10] also demonstrated similar trends during the countermovement shrug at 140% 1RM PC, in which the first repetition was not always the fastest, and during the RR_45_ protocol, the second repetition was always faster than the first. Therefore, it appears that utilising RR to perform individual repetitions may not be suitable if the aim is to maximise acute performance, as the first repetition following a rest period likely displays a reduction in performance compared to subsequent repetitions until fatigue ensues [30]. When investigating the effects of RR and TS on the clean pull, strength-trained males achieved greater vertical barbell displacement with RR compared with TS [5]. The differences observed in this study (S1 Fig) demonstrated that there is likely repetition to repetition variations, despite verbal encouragement, and provides support for the effectiveness of RR compared with TS, as provides a more frequent opportunity for feedback, which may enhance the overall kinetic and kinematic output, and further investigation in this area should be conducted.

Rating of perceived exertion across entire set-protocols was also not different in RR protocols compared to TS. This is not surprising given that there were no differences between TS and RR configurations on kinetics or kinematics, except for significantly greater PV during the RR_72_ compared to RR_45_, although the highest RPE was reported during the TS, and lowest during the RR_72_. Although there are minimum differences in kinetics and kinematics between sets, there were significant differences between all sets for RPE in all configurations. Practitioners should be mindful of this when prescribing supramaximal HP throughout different phases of a training cycle due to the increasing RPE value, particularly during key competition phases. Our results indicate that despite the TS protocol having greater RPE than RR protocols (Tables 1 and 3), RR resulted in similar PF, MF, MV, IMP, propulsion duration, PVM, and RPE across multiple sets, which may be due to the reduced range of motion of the HP, compared to exercises such as the clean pull, in which RR appeared to be better at maintaining kinetics and kinematics compared to TS structures [4]. Jukic and Tufano [5] reported significantly lower RPE during the RR protocols compared to TS regardless of whether RR or TS configurations were performed, during the clean pull from the floor, and showed that RPE increased across sets at all loads, demonstrating that RR was perceptually easier compared to TS. Jukic et al. [4, 5] also reported greater RPE (8.04 ± 1.16) and (7.77 ±1.15) at 120% 1RM PC during the TS protocol, compared to (7.07 ± 1.03) at 140% 1RM PC (Table 1), and this is likely explained by the greater ROM performed during the clean pull from the floor compared to the HP.

Moderate to large differences were observed between sets for RPE for TS, RR_45_ and RR_72_ (Table 3) and showed a progressively greater RPE with an increase in sets, and therefore total repetitions in line with previous research during TS, albeit during the PC [9]. This increase was not consistently evident in the kinetics and kinematics variables, with set 3 eliciting the peak values in many variables (Table 3). This potentially questions the appropriateness of utilising RPE scores to represent changes in kinetic and kinematics due to the minimal meaningful or significant differences observed (Tables 1 and 3; S3A and S3B Fig).

The findings of this study are not without their limitations. Whilst this study and previous studies have investigated force-time characteristics of weightlifting pulling derivatives using loads that have been calculated from the 1RM of a weightlifting catching variation [1, 2, 4–6], the authors acknowledge that it likely impractical to perform 1RM tests for certain pulling derivatives. Given the multiple sport cohort in this study, these findings should be interpreted with caution as assessing competitive weightlifters may yield different conclusions. Caution is needed when comparing these results to other studies as the HP utilises the SSC and is a semi-ballistic task which requires maximal intent, whereas squats and bench press include a substantial deceleration phase [31] and may not be conducive to perform with a comparable ballistic intent.

## Conclusions

The study’s findings indicated that the absence of significant differences in velocity and force output (including power, most likely) could be attributed to the limited fatigue experienced during TS, possibly because of the minimal displacement of the HP. Implementing RR periods to create shorter, but more frequent sets may only result in improved maintenance of kinetic and kinematics when a comparative TS structure is highly fatiguing, or when a larger range of motion is performed, (e.g., clean pulls) [6]. The findings of the study show that practitioners can perform various set and repetition configurations at loads of 140% 1RM without having a negative impact on kinetics and kinematics, which may provide a unique strength-speed’ stimulus. Having athletes perform RR protocols will allow practitioners opportunity to provide more frequent technical feedback, but careful consideration should be paid to the frequency of un-racking and re-racking the barbell, particularly at heavy loads (>85% 1RM) as this may contribute to overall fatigue and affect resistance training performance [11], and a greater understanding of the acute effects of intra-set rest periods may improve subsequent program design.

## Supporting information

S1 Fig **a-f:** Mean and standard deviation across 18 repetitions for the hang pull at 140% 1RM PC for traditional sets (Black circles), Rest Redistribution (Open circles) with 45s inter-repetition rest (RR_45_) and 72s inter-repetition rest (Black triangles) (RR_72_).
(DOCX)

S2 Fig a: Maintenance of peak velocity for 3 sets of 6 repetitions and b) Maintenance of peak velocity for all entire protocols.
(DOCX)

S3 Fig a: Rating of perceived exertion for all 3 sets of 6 repetitions and b) Rating of perceived exertion for each set protocol across all repetitions.
(DOCX)

## References

[1] Meechan D, McMahon JJ, Suchomel TJ, Comfort P. A Comparison of Kinetic and Kinematic Variables During the Pull From the Knee and Hang Pull, Across Loads. J Strength Cond Res. 2020;34(7):1819–29. 10.1519/JSC.0000000000003593 32282627

[2] Meechan D, Suchomel TJ, McMahon JJ, Comfort P. A Comparison of Kinetic and Kinematic Variables During The Mid-Thigh Pull and Countermovement Shrug, Across Loads. J Strength Cond Res. 2020;34(7):1830–41. doi: 10.1519/JSC.0000000000003288 32358309

[3] Suchomel TJ, Comfort P, Stone MH. Weightlifting Pulling Derivatives: Rationale for Implementation and Application. Sports Med. 2015;45(6):823–39. doi: 10.1007/s40279-015-0314-y 25689955

[4] Jukic I, Tufano JJ. Rest Redistribution Functions as a Free and Ad-Hoc Equivalent to Commonly Used Velocity-Based Training Thresholds During Clean Pulls at Different Loads. Journal of Human Kinetics. 2019;68:5–16. doi: 10.2478/hukin-2019-0052 31531129 PMC6724594

[5] Jukic I, Tufano JJ. Acute effects of shorter but more frequent rest periods on mechanical and perceptual fatigue during a weightlifting derivative at different loads in strength-trained men. Sports Biomech. 2020:1–14. Epub 2020/04/28. doi: 10.1080/14763141.2020.1747530 . 32336216

[6] Haff GG, Whitley A, McCoy LB, O’Bryant HS, Kilgore JL, Haff EE, et al. Effects of different set configurations on barbell velocity and displacement during a clean pull. J Strength Cond Res. 2003;17(1):95–103. doi: 10.1519/1533-4287(2003)017&lt;0095:eodsco&gt;2.0.co;2 12580663

[7] Hardee JP, Triplett NT, Utter AC, Zwetsloot KA, McBride JM. Effect of interrepetition rest on power output in the power clean. J Strength Cond Res. 2012;26(4):883–9. doi: 10.1519/JSC.0b013e3182474370 . 22228112

[8] Hardee JP, Lawrence MM, Zwetsloot KA, Triplett NT, Utter AC, McBride JM. Effect of cluster set configurations on power clean technique. J Sports Sci. 2013;31(5):488–96. doi: 10.1080/02640414.2012.736633 . 23121475

[9] Hardee JP, Lawrence MM, Utter AC, Triplett NT, Zwetsloot KA, McBride JM. Effect of inter-repetition rest on ratings of perceived exertion during multiple sets of the power clean. Eur J Appl Physiol. 2012;112(8):3141–7. doi: 10.1007/s00421-011-2300-x . 22215288

[10] Meechan D, McMahon JJ, Suchomel TJ, Comfort P. The Effect of Rest Redistribution on Kinetic and Kinematic Variables During the Countermovement Shrug. Journal Strength Cond Res. 2023;37(7):1358–66. doi: 10.1519/JSC.0000000000004238 37347941

[11] Jukic I, Ramos AG, Helms ER, McGuigan MR, Tufano JJ. Acute Effects of Cluster and Rest Redistribution Set Structures on Mechanical, Metabolic, and Perceptual Fatigue During and After Resistance Training: A Systematic Review and Meta-analysis. Sports Med. 2020;50(12):2209–36. Epub 2020/09/10. doi: 10.1007/s40279-020-01344-2 . 32901442

[12] Robertson RJ, Goss FL, Rutkowski J, Lenz B, Dixon C, Timmer J, et al. Concurrent validation of the OMNI perceived exertion scale for resistance exercise. Med Sci Sports Exerc. 2003;35(2):333–41. Epub 2003/02/06. doi: 10.1249/01.MSS.0000048831.15016.2A . 12569225

[13] Tufano JJ, Conlon JA, Nimphius S, Brown LE, Banyard HG, Williamson BD, et al. Cluster Sets: Permitting Greater Mechanical Stress Without Decreasing Relative Velocity. Int J Sports Physiol Perform. 2017;12:463–9. doi: 10.1123/ijspp.2015-0738 27617387

[14] Owen N, Watkins J, Kilduff L, Bevan H, Bennett M. Development of a criterion method to determine peak mechanical power output in a countermovement jump. J Strength Cond Res. 2014;28(6):1152–8. doi: 10.1519/JSC.0000000000000311 24276298

[15] McMahon JJ, Suchomel TJ, Lake J, Comfort P. Understanding the Key Phases of the Countermovement Jump Force-Time Curve. Strength Cond J. 2018;40:96–106. doi: 10.1519/SSC.0000000000000375

[16] McMahon JJ, Jones PA, Dos’ Santos T, Comfort P. Influence of Dynamic Strength Index on Countermovement Jump Force-, Power-, Velocity-, and Displacement-Time Curves. Sports. 2017;5(72):1–11. doi: 10.3390/sports5040072 29910432 PMC5969025

[17] McMahon JJ, Murphy S, Rej SJE, Comfort P. Countermovement Jump-Phase Characteristics of Senior and Academy Rugby League Players. Int J Sports Physiol Perform. 2017;12(6):803–11. Epub 2016/12/06. doi: 10.1123/ijspp.2016-0467 . 27918658

[18] McMahon JJ, Rej SJE, Comfort P. Sex Differences in Countermovement Jump Phase Characteristics. Sports (Basel, Switzerland). 2017;5(1):1–11. doi: 10.3390/sports5010008 . 29910368 PMC5969005

[19] Koo TK, Li MY. A Guideline of Selecting and Reporting Intraclass Correlation Coefficients for Reliability Research. J Chiropr Med. 2016;15(2):155–63. doi: 10.1016/j.jcm.2016.02.012 . 27330520 PMC4913118

[20] Cormack SJ, Newton RU, McGuigan MR, Doyle TLA. Reliability of Measures Obtained During Single and Repeated Countermovement Jumps. Int J Sports Physiol Perform. 2008;3:131–44. doi: 10.1123/ijspp.3.2.131 19208922

[21] Hopkins WG, Marshall SW, Batterham AM, Hanin J. Progressive Statistics for Studies in Sports Medicine and Exercise Science. Med Sci Sports 2009;41(1):3–12. doi: 10.1249/MSS.0b013e31818cb278 19092709

[22] Turner AN, Comfort P, McMahon J, Bishop C, Chavda S, Read P, et al. Developing Powerful Athletes Part 2: Practical Applications. Strength Cond J 2021;43(1):23–31.

[23] Haff GG, Nimphius S. Training Principles for Power. Strength Cond J. 2012;34(6):2–12.

[24] Suchomel TJ, Comfort P, Lake JP. Enhancing the force-velocity profile of athletes using weightlifting derivatives. Strength Cond J. 2017;39(1):10–20.

[25] Lake J, Lauder M, Smith N. Barbell kinematics should not be used to estimate power output applied to the barbell- and-body system center of mass during lower-body resistance exercise. J Strength Cond Res. 2012;26(5):1302–7. doi: 10.1519/JSC.0b013e31822e7b48 22516904

[26] García Ramos A. Resistance training intensity prescription methods based on lifting velocity monitoring. Int J Sports Med. 2023. Epub 2023/08/23. doi: 10.1055/a-2158-3848 . 37607576

[27] Oliver JM, Kreutzer A, Jenke SC, Phillips MD, Mitchell JB, Jones MT. Velocity drives greater power observed during back squat using cluster sets. J Strength Cond Res. 2016;30(1):235–43. doi: 10.1519/JSC.0000000000001023 26121432

[28] Tufano JJ, Conlon JA, Nimphius S, Brown LE, Seitz LB, Williamson BD, et al. Maintenance of Velocity and Power With Cluster Sets During High-Volume Back Squats. Int J Sports Physiol Perform. 2016;11:885–92. doi: 10.1123/ijspp.2015-0602 26791936

[29] de-Oliveira LA, Heredia-Elvar JR, Maté-Muñoz JL, García-Manso JM, Aragão-Santos JC, Da Silva-Grigoletto ME. Analysis of Pacing Strategies in AMRAP, EMOM, and FOR TIME Training Models during "Cross" Modalities. Sports (Basel, Switzerland). 2021;9(11). doi: 10.3390/sports9110144 . 34822344 PMC8624389

[30] Merrigan JJ, Jones MT, Padecky J, Malecek J, Omcirk D, Scott BR, et al. Impact of Rest-Redistribution on Fatigue During Maximal Eccentric Knee Extensions. J Hum Kinet. 2020;74:205–14. doi: 10.2478/hukin-2020-0028 . 33312288 PMC7706641

[31] Elliott BC, Wilson GJ, Kerr GK. A biomechanical analysis of the sticking region in the bench press. Med Sci Sports Exerc. 1989;21(4):450–62. Epub 1989/08/01. . 2779404

