# Supplementary material for: The effect of rest redistribution on kinetic and kinematic variables during the hang pull
Source: PLoS One. 2024 Feb 26;19(2):e0299311. doi: 10.1371/journal.pone.0299311 (PMC10896527; doi:10.1371/journal.pone.0299311)
Supplement: S1 Fig — a-f: Mean and standard deviation across 18 repetitions for the hang pull at 140% 1RM PC for traditional sets (Black circles), Rest Redistribution (Open circles) with 45s inter-repetition rest (RR45) and 72s inter-repetition rest (Black triangles) (RR72). (DOCX) [file pone.0299311.s001.docx]

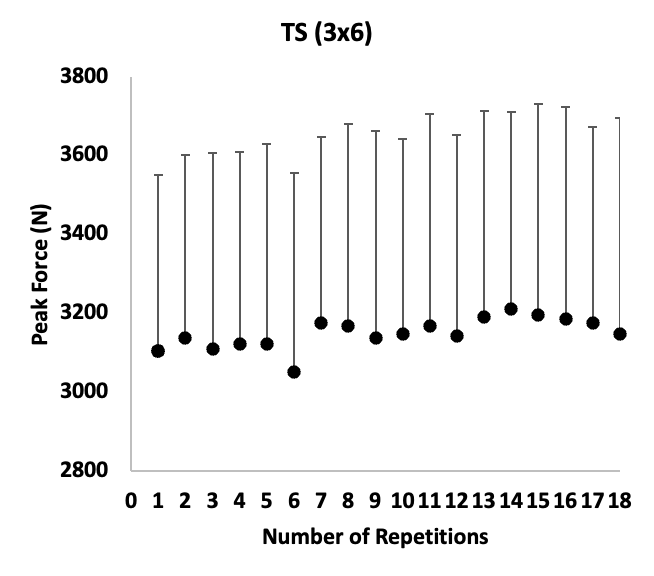

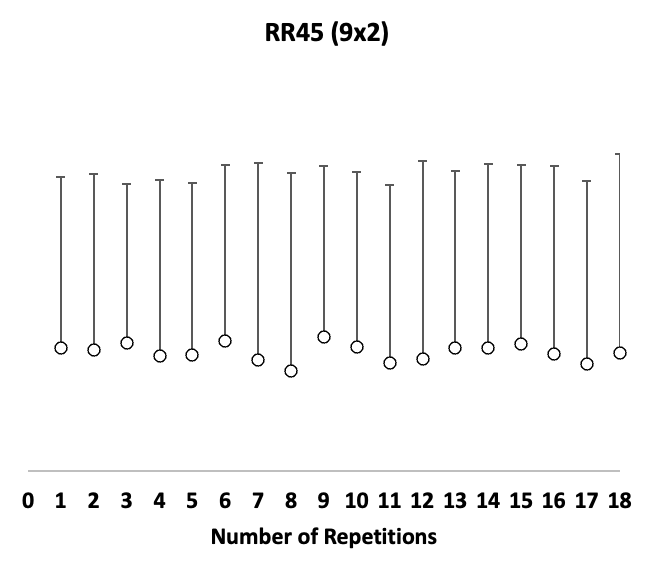

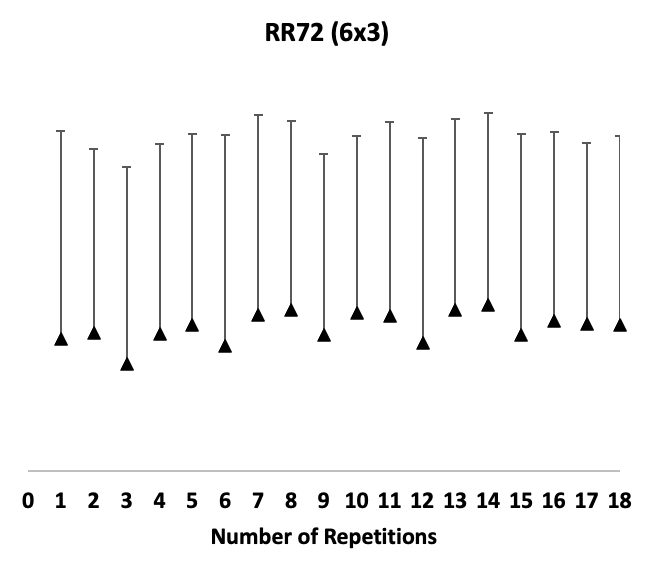


**a)**

No significant (p>0.05) differences between protocols across the average of 18 repetitions

**b)**


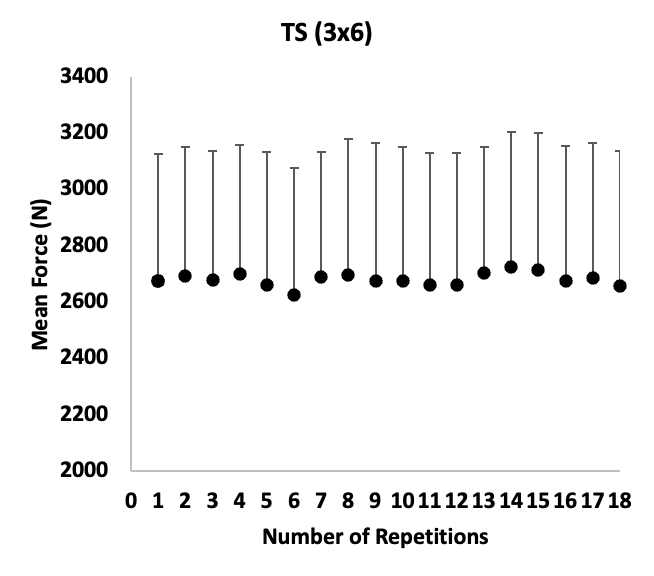

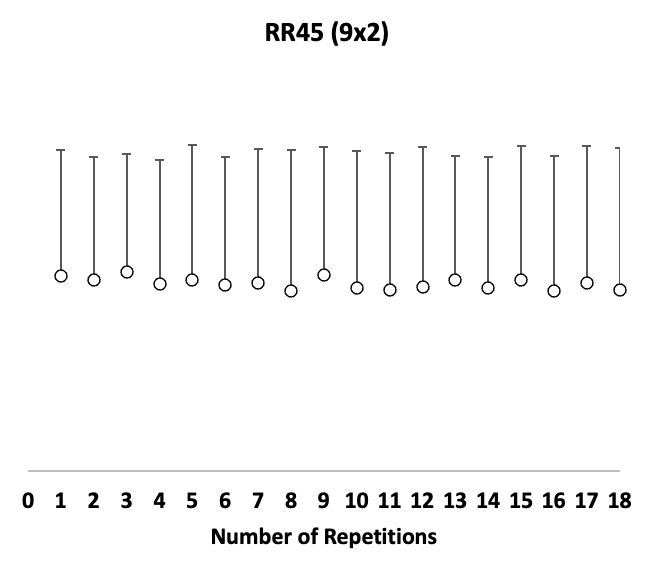

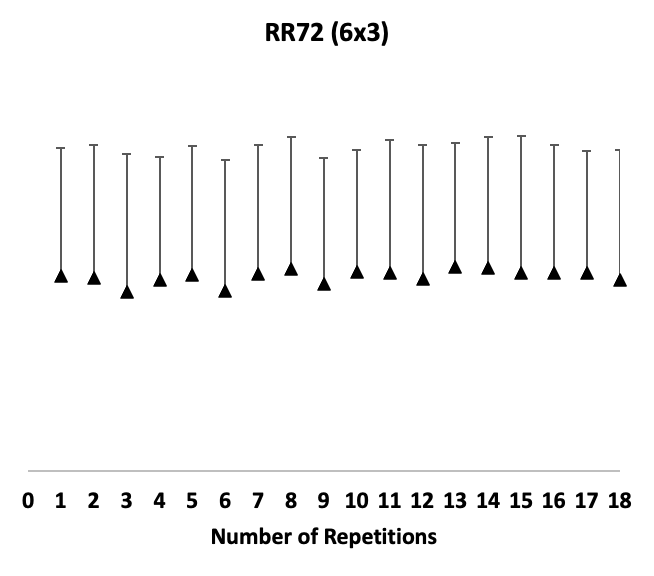


No significant (*p* > 0.05) differences between protocols across the average of 18 repetitions


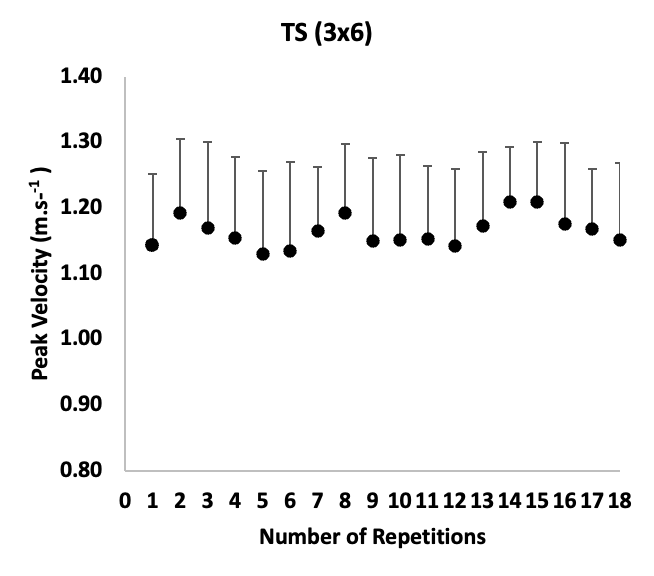

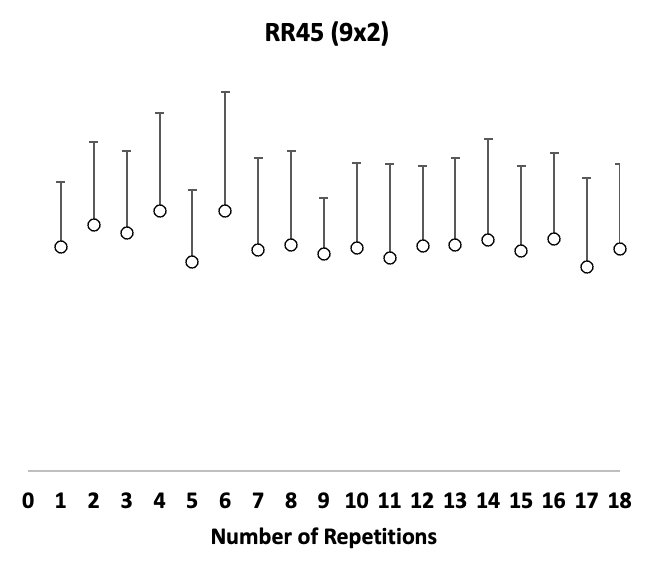

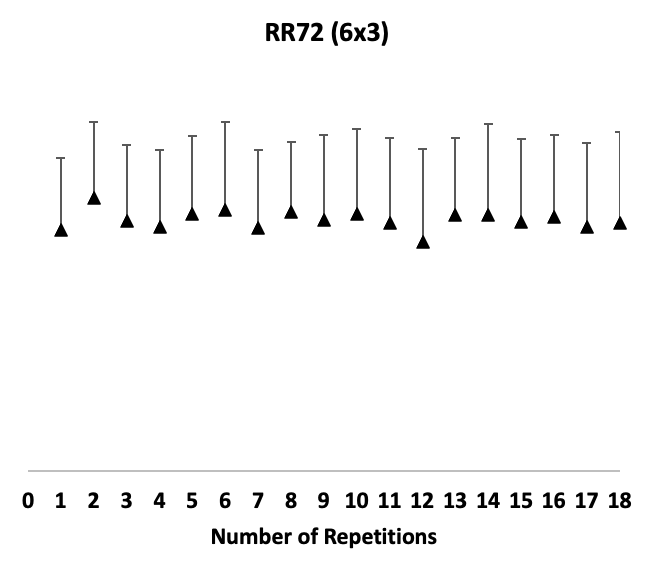


**c)**

**＊**

＊Significantly (*p* = 0.025) greater PV for RR_72_ compared to RR_45_ for configuration

**d)**


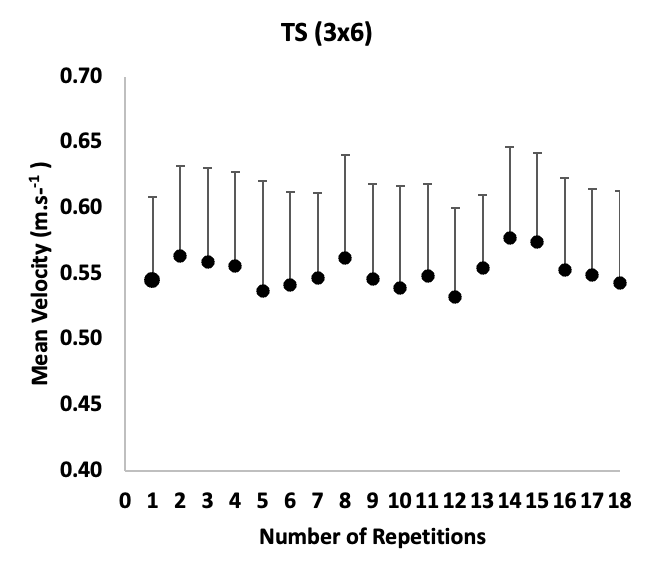

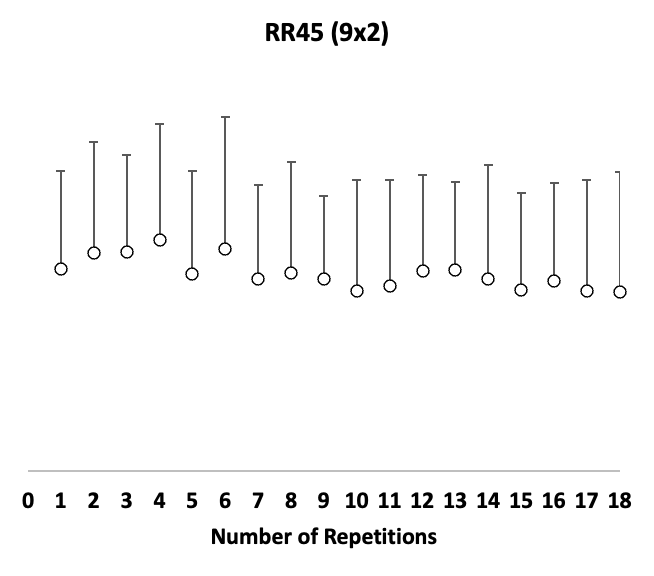

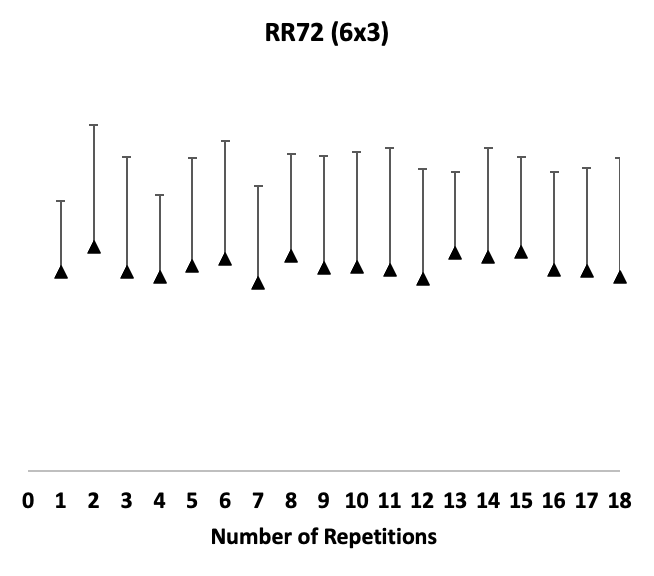


No significant (*p* > 0.05) differences between protocols across the average of 18 repetitions


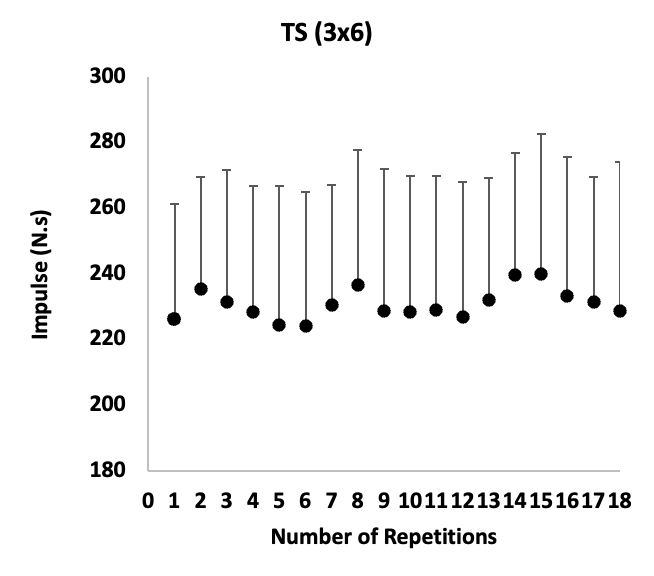

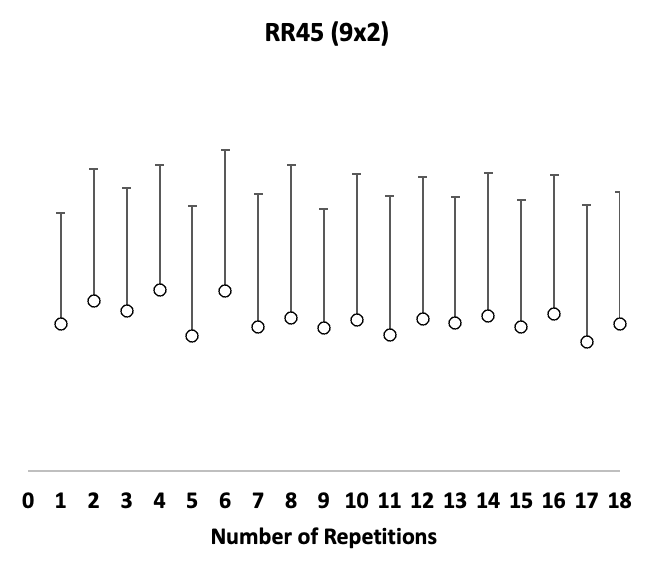

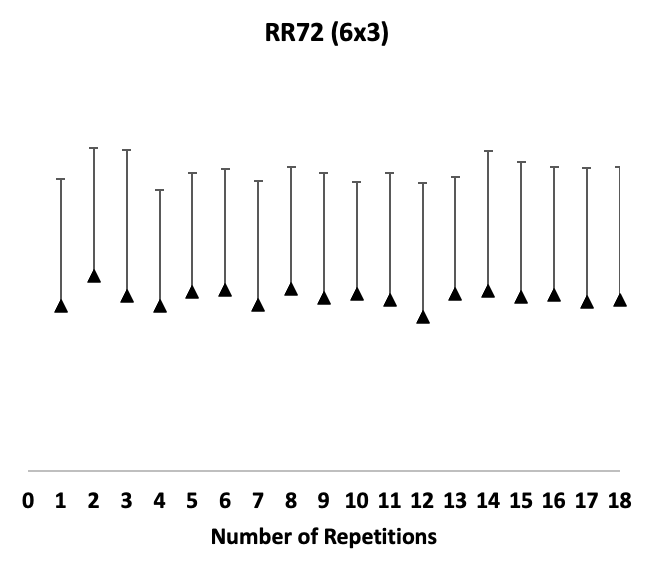


**e)**

No significant (p *>* 0.05) differences between protocols across the average of 18 repetitions

**f)**


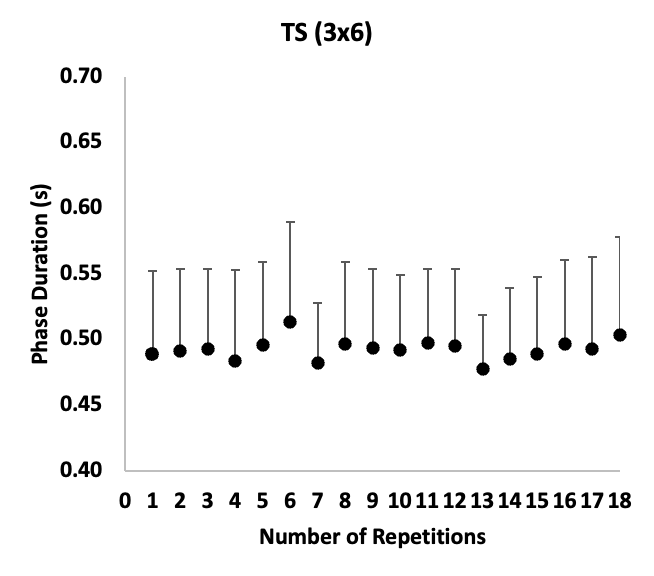

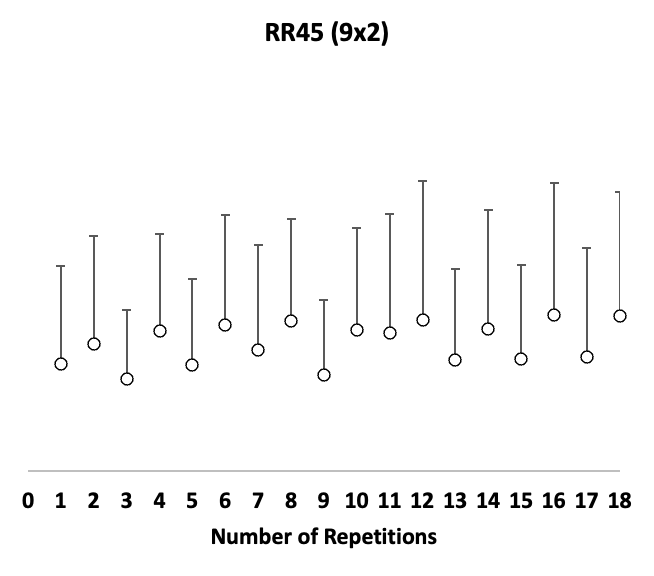

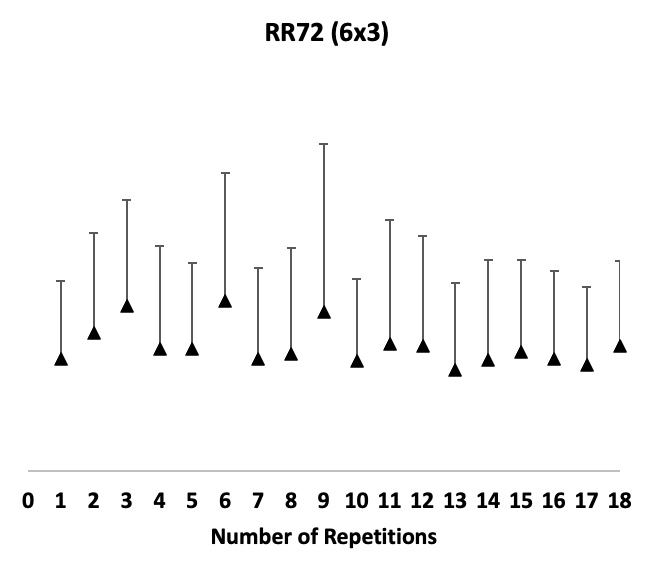


No significant (*p* > 0.05) differences between protocols across the average of 18 repetitions

**Fig 3**- Mean and standard deviation across 18 repetitions for the hang pull at 140% 1RM PC for traditional sets (black circles), rest redistribution (open circles) with 45s inter-repetition rest (RR_45_) and 72s inter-repetition rest (black triangles) (RR_72_).
