# Supplementary material for: The effect of rest redistribution on kinetic and kinematic variables during the hang pull
Source: PLoS One. 2024 Feb 26;19(2):e0299311. doi: 10.1371/journal.pone.0299311 (PMC10896527; doi:10.1371/journal.pone.0299311)
Supplement: S2 Fig — a: Maintenance of peak velocity for 3 sets of 6 repetitions and b) Maintenance of peak velocity for all entire protocols. (DOCX) [file pone.0299311.s002.docx]

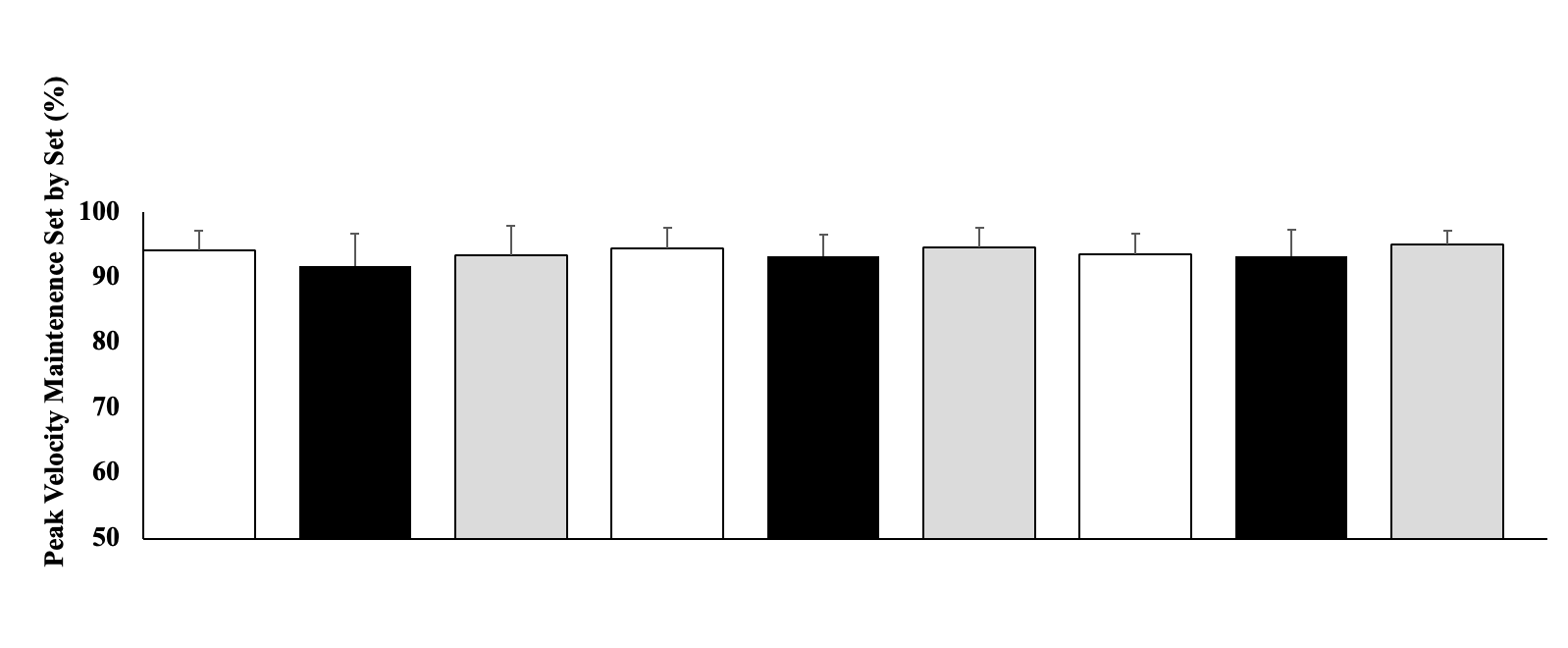

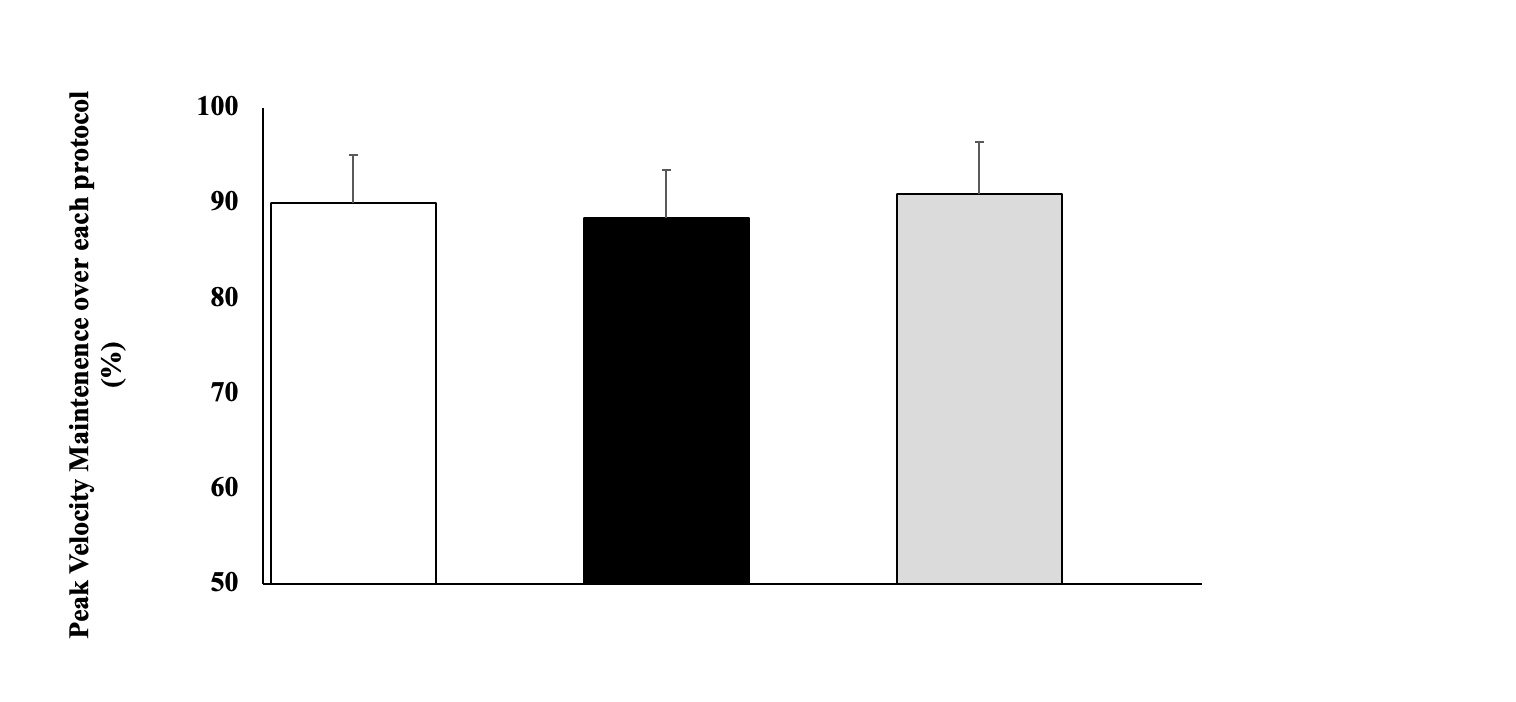


**TS**

**RR_45_**

**RR_72_**

**TS RR_45_ RR_72_**

**Set 1 Set 2 Set 3**

a)

b)

**Fig 4a**) Maintenance of peak velocity for 3 sets of 6 repetitions **4b)** Maintenance of peak velocity for all entire protocols
