# Supplementary material for: The effect of rest redistribution on kinetic and kinematic variables during the hang pull
Source: PLoS One. 2024 Feb 26;19(2):e0299311. doi: 10.1371/journal.pone.0299311 (PMC10896527; doi:10.1371/journal.pone.0299311)
Supplement: S3 Fig — a: Rating of perceived exertion for all 3 sets of 6 repetitions and b) Rating of perceived exertion for each set protocol across all repetitions. (DOCX) [file pone.0299311.s003.docx]

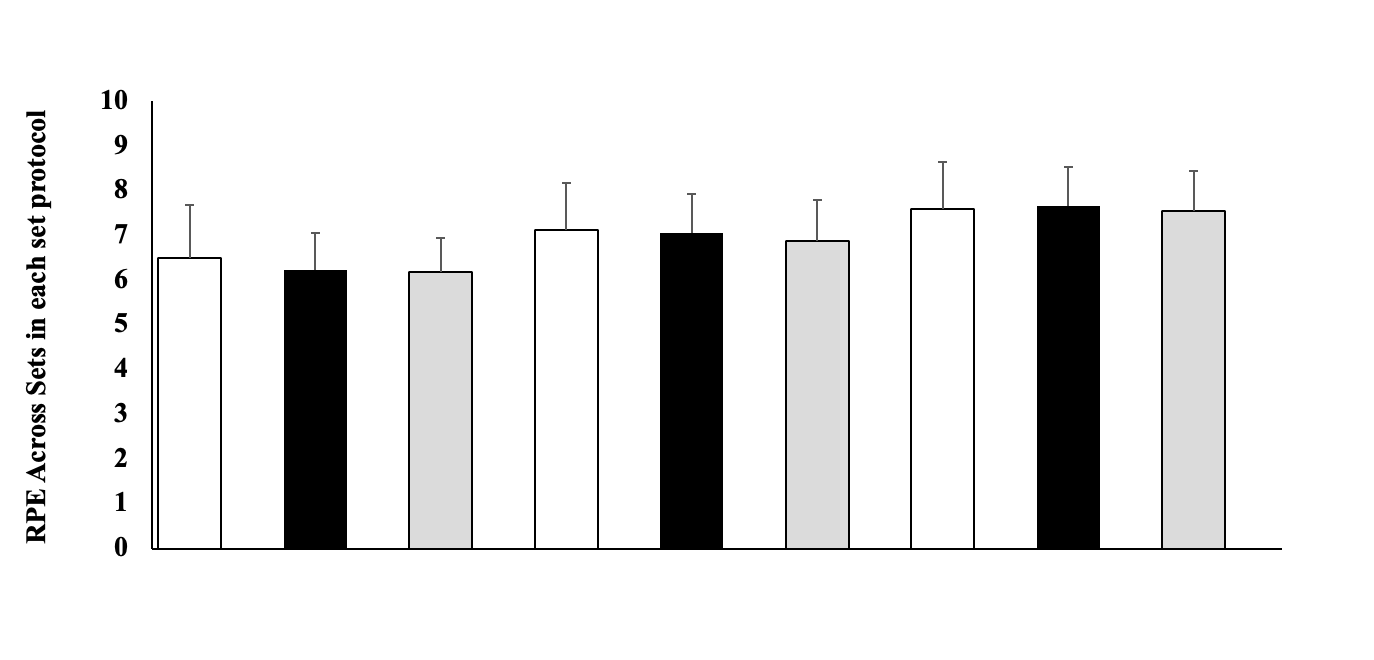

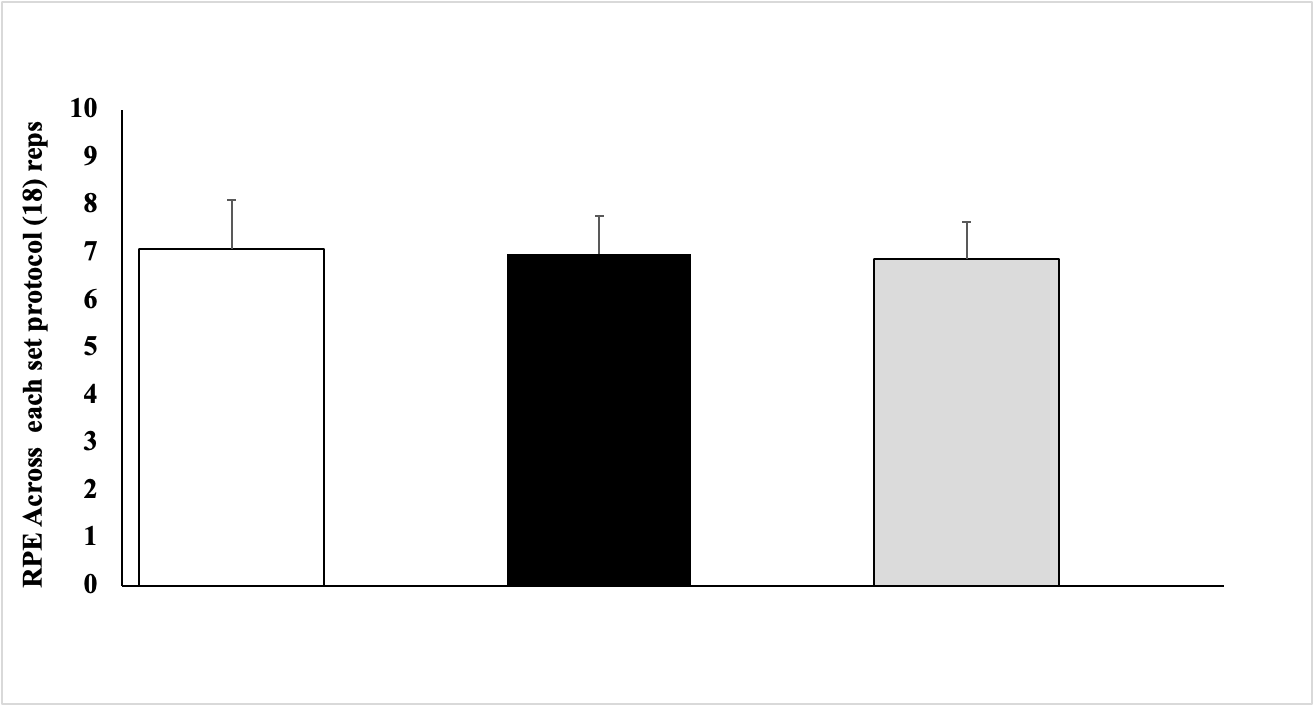


*

**Set 1 Set 2 Set 3**

**TS RR_45_ RR_72_**

**TS**

**RR_45_**

**RR_72_**

##

b)

a)

**Fig 5a**) RPE for all collapsed 3 sets of 6 repetitions **5b)** RPE for each set protocol across all repetitions

* Significantly greater (*p* ≤0.001) than Set 1

## Significantly greater (*p* ≤ 0.015) than Set 1 & 2
